# Biological Function of Antimicrobial Peptides on Suppressing Pathogens and Improving Host Immunity

**DOI:** 10.3390/antibiotics12061037

**Published:** 2023-06-10

**Authors:** Zhiqian Lyu, Pan Yang, Jian Lei, Jinbiao Zhao

**Affiliations:** 1 Guangdong Haid Group Co., Ltd., Guangzhou 511400, China; lvzq@haid.com.cn (Z.L.); leij01@haid.com.cn (J.L.); 2 State Key Laboratory of Animal Nutrition, College of Animal Science and Technology, China Agricultural University, Beijing 100193, China; ypan23@cau.edu.cn; 3 Qingyuan Haibei BIO-TECH Co., Ltd., Qingyuan 511853, China

**Keywords:** Antimicrobial peptides, antibiotics alternatives, physical and chemical properties, immune function, host health

## Abstract

The emergence of drug-resistant genes and concerns about food safety caused by the overuse of antibiotics are becoming increasingly prominent. There is an urgent need for effective alternatives to antibiotics in the fields of livestock production and human medicine. Antimicrobial peptides can effectively replace antibiotics to kill pathogens and enhance the immune functions of the host, and pathogens cannot easily produce genes that are resistant to them. The ability of antimicrobial peptides (AMPs) to kill pathogens is associated with their structure and physicochemical properties, such as their conformation, electrical charges, hydrophilicity, and hydrophobicity. AMPs regulate the activity of immunological cells and stimulate the secretion of inflammatory cytokines via the activation of the NF-κB and MAPK signaling pathways. However, there are still some limitations to the application of AMPs in the fields of livestock production and human medicine, including a restricted source base, high costs of purification and expression, and the instability of the intestines of animals and humans. This review summarizes the information on AMPs as effective antibiotic substitutes to improve the immunological functions of the host through suppressing pathogens and regulating inflammatory responses. Potential challenges for the commercial application of AMPs in animal husbandry and human medicine are discussed.

## 1. Introduction

In recent years, an increasing number of people have gradually realized problems with food safety, the emergence of drug-resistant super-bacteria, and environmental pollution caused by the abuse of antibiotics [1]. Many researchers have reported that antimicrobial peptides (AMPs) suppress the growth of pathogens, strengthen the immunological functions of the host, and improve intestinal health [2,3]. AMPs function to kill pathogens via a variety of mechanisms, such as the destruction of cell walls by binding to peptidoglycans and the perforation of cell membranes by linking to phospholipids, interfering with the reverse transcription of RNA, and activating host immunological systems [4]. Recently, many researchers have shown that AMPs, especially bacteriocin, can regulate gut microbiota to secrete quorum-sensing signal molecules, which suppress the production of endotoxins from pathogens and activate the mTOR pathways of intestinal epithelial cells to enhance the intestinal barrier function [5,6]. The extensive application of AMPs is unlikely to produce resistant genes in gut bacteria [7,8]. Therefore, AMPs are one of the most effective substitutes for antibiotics in human medicine and livestock production. There is increasing interest in the potential of AMPs to partially or completely replace antibiotics. However, the potential mechanisms of AMPs with respect to host immunology, such as interactions between the molecular structure of AMPs and their biological activity against bacteria and their effects on the host’s immune system, are unclear.

The application of AMPs in practice is severely limited due to their small scale of production and high purification cost. Normally, AMPs are encoded by gene clusters most often carried by plasmids and introduced into prokaryotic or eukaryotic expression systems to achieve commercial production [9,10]. Prokaryotic expression yields the biological activity of AMPs due to their lack of intracellular modifications and secretion of endotoxins. Eukaryotic expression systems explain the low expression efficiency and high expression cost of AMPs.

This review summarizes the molecular structure, physical properties, advances in host immunological functions, and advantages and disadvantages of biological expression systems for AMPs. The goal of this field is to find effective alternatives to antibiotics for use in human health, animal production, and disease control.

## 2. Definition of AMPs

AMPs are a class of peptides that widely exist in nature and they are an important part of the innate immune system of different organisms. AMPs have a wide range of inhibitory effects against bacteria, fungi, parasites, and viruses [11]. Most AMPs have a strong capacity to resist acidity and alkalinity, are thermally stable, and have a wide spectrum of antibacterial functions. Normally, they cannot resist proteases in the small intestine of the host that hydrolyze and inactivate AMPs [12]. AMPs play an important role in modulating the natural defense systems of the host, such as resisting adhesion and colonization by harmful bacteria [13].

At present, the term AMPs includes all AMPs from all organisms and microorganisms (eukaryotes and prokaryotes) and from the two biosynthetic pathways (ribosome-independent and ribosome-dependent pathways), and AMPs from superior organisms are preferentially termed host defense peptides (HDPs) [14,15,16,17]. In recent years, however, researchers have discovered that AMPs secreted by bacteria or post-translational modifications such as bacteriocins are usually more effective in suppressing colonization by harmful bacteria than those produced by eukaryotes [18].

## 3. Properties and Structure of AMPs

Although there are many sources of AMPs, some physical characteristics are common among the sources. Firstly, AMPs usually have relatively short sequences consisting of 12 to 50 amino acids with positive charges and contain a high proportion of hydrophobic amino acid residues [19]. AMPs usually carry a net positive charge ranging from +2 to +13 and have specific cationic domains. The cationic properties of AMPs, which directly influence their antibacterial activity, are associated with the presence of lysine and arginine residues [20]. Hydrophobicity is a percentage of hydrophobic amino acid residues in a peptide sequence. The degree of distribution of water-soluble peptides in the membrane lipid bilayer is limited by hydrophobicity, being related to membrane permeation. However, the high hydrophobicity of AMPs leads to their cytotoxicity to mammalian cells and decreases the activity of antibacterials [21]. In addition, AMPs have both hydrophilic and hydrophobic properties. Amphiphilicity refers to the relative abundance of hydrophilic and hydrophobic domains, which is considered to be a balance between cations and hydrophobic residues [22,23].

**Figure 1:**
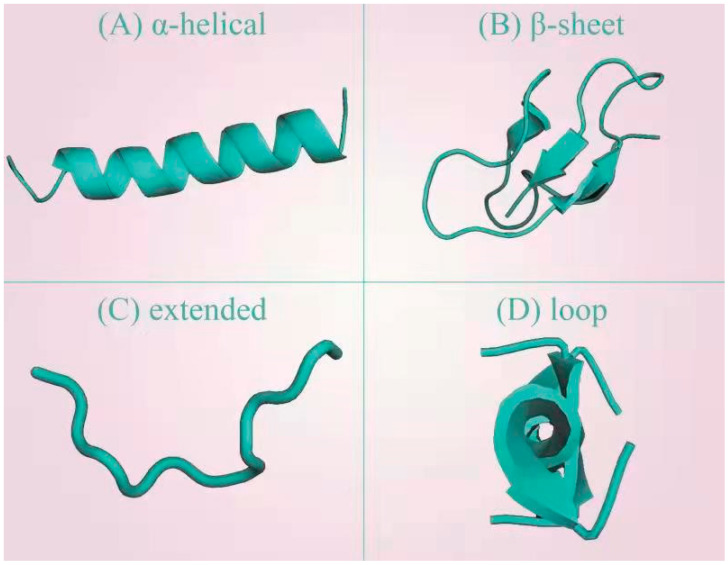
Modes of protein structures in different AMPs. (**A**) The α-helical structure (DOI: 10.2210/pdb2K6O/pdb). α-helical peptides are some of the most abundant and widespread AMPs and appear to represent a successful structural arrangement in natural defense systems. They tend to be short (<40 amino acid residues) and are suited to chemical synthesis. (**B**) The β-sheet structure (DOI: 10.2210/pdb1KFP/pdb). The β-sheet structure of AMPs is common in humans and animals, such as human defense peptides. (**C**) Extended structure. The typical representative of the extended structure is indolicidin (DOI: 10.2210/pdb1G89/pdb), which always contains abundant tryptophan and glycine. (**D**) Loop structure. The primary looped structure of AMPs is bactenecin (DOI: 10.2210/pdb2plh/pdb), which contains an intramolecular covalent disulfide bond on its C-end, resulting in a loop.

Correctly, four different three-dimensional structures of AMPs have been reported, including the beta-sheet structure with disulfide bonds and alpha-helical, extended, and looped structures [24,25,26]. Protein models representing structural differences between the four types of AMPs are shown in Figure 1 [27]. Alpha-helical cathelicidian and beta-sheet defensin structures are common in humans. The typical representative of the extended structure is indolicidin, which always contains ample tryptophan and glycine. The looped structure of AMPs of bactenecin contains an intramolecular covalent disulfide bond on its C-end, which results in a loop. In addition, there are a large number of AMPs, such as human lactoferricin, whose structure is a mixture of the common structures mentioned above.

## 4. Comparison between AMPs and Antibiotics

Traditionally, antibiotics have played important roles in controlling human disease, ensuring food security, and enhancing livestock production. However, the emergence and spread of bacterial resistance caused by the excessive use of antibiotics seriously threatens human health [28]. A comparison of conventional antibiotics with AMPs is presented in Table 1 [29]. A difference between bacteriocin and antibiotics synthesized via secondary metabolic pathways is that bacteriocins are polypeptides and proteins produced on the bacterial ribosome. AMPs use numerous mechanisms to kill harmful bacteria, fungi, and viruses. In addition, traditional antibiotics have a broad spectrum of activity, but easily promote the development of resistance to antibiotics among pathogens [28]. In addition to direct antibacterial activity, AMPs promote the gut homeostasis and health of the host by regulating immunological functions and gut microbial communities. AMPs are interesting candidates for the replacement of traditional antibiotics in suppressing some harmful bacteria that are easily resistant to traditional antibiotics. A novel antimicrobial peptide polymer, G3KL, has a positive effect in suppressing the multi-drug-resistant strains of *Acinetobacter baumannii* and *Pseudomonas aeruginosa* [30]. An antimicrobial peptide derived from *Chiromyothiasis* shows a strong effect against antibiotic-resistant *Staphylococcus aureus* by destroying the cell membrane of the targeted pathogen [31].

## 5. Synthesis of AMPs

Generally, natural AMPs are synthesized through two different pathways: ribosome-independent and ribosome-dependent. AMPs produced through the ribosome-dependent pathway primarily originate from insects, plants, mammals, and some microorganisms.

### 5.1. Defensins

Defensins are cationic polypeptides with many disulfide bonds, which are widely distributed in fungi, plants, and animals, and they play a vital role in modulating the immunological functions of the host [32,33]. Defensins can be divided into mammalian, insect, and plant defensins. Most defensins are composed of 29 to 52 amino acid residues and contain three pairs of intramolecular disulfide bonds with a relative molecular weight of 2 to 6 kDa. They are divided into α-, β-, and θ-defensins in reference to the positions of the disulfide bonds [34]. The positions of the disulfide bonds in the α-defensin molecular chain are Cys1–Cys6, Cys2–Cys4, and Cys3–Cys5, where Cys1–Cys6 connects the N and C ends to form a circular molecule. The positions of the disulfide bonds in the β-defensin molecular chain are Cys1–Cys5, Cys2–Cys4, and Cys3–Cys6. The positions of the disulfide bonds in the θ-defensin molecular chain are Cys1–Cys6, Cys2–cys5, and Cys3–Cys4, which are comprised of the three disulfide bonds mentioned above [35]. Most defensins derived from insects have a net positive charge and are composed of 34 to 51 amino acids with a molecular weight of 4 kDa [36]. The amino acid sequences contain six conserved cysteine residues. β-sheet and α-helix are stabilized by disulfide bonds connected by Cys1–Cys4, Cys2–Cys5, and Cys3–Cys6. The disulfide bonds and three-dimensional spatial conformation of insect defensins and mammal defensins are quite different, which proves that insect defensins and mammal defensins are not homologous. The molecular weight of plant defensins is less than 5 kDa, and they are composed of 45 to 54 amino acids [37]. The disulfide bonds of plant defensins form a reverse β-sheet structure and an α-helix structure, contain four pairs of disulfide bonds, and are connected in the following ways: Cys1–Cys8, Cys2–Cys5, Cys3–Cys6, and Cys4–Cys7.

### 5.2. Bacteriocin

Bacteriocins are synthesized by bacteria to kill microorganisms within a selective and narrow spectrum [38]. Bacteriocins are divided into four groups, including lantibiotics, small heat-stable peptides, large heat-labile peptides, and complex peptides combined with carbohydrate or lipid groups [39]. Lantibiotics are comprised of 19 to 50 amino acids and are characterized by some rare amino acids, such as lanthionine, β-methyllanthionine, and dehydrobutyrine, which are located in their active molecular zones [40]. Small heat-stable peptides with a molecular weight of less than 10 kDa are produced by some bacteria and are not modified. Large heat-labile proteins with a molecular weight of more than 10 kDa have a narrow spectrum of antimicrobial activity and lose functionality after heating for 30 s at 100 ℃. Complex peptides are macromolecules containing carbohydrate or lipid groups in addition to amino acids, which easily form complexes when combined with other organic substrates of the crude extract due to the positive charge and hydrophobicity of bacteriocins. The modification of specific amino acids and chemical structures of bacteriocins normally improve their molecular stability, extend their half-life, and expand their antimicrobial spectrum of activity [41,42].

### 5.3. Antimicrobial Substrates Produced in a Ribosome-Independent Way

Unlike AMPs and active biological enzymes synthesized by a ribosome-dependent pathway, the synthesis of antimicrobial substrates depending on non-ribosomal peptide synthetase are always produced by bacteria, such as *Bacillus*, which include lipopeptides, glycopeptides, and linear polyamides. Non-ribosomal peptides are synthesized through multi-functional enzyme systems by sulfur-template polymerase and are further modified through methylation, acylation, glycosylation, heterocyclization, and cyclization to exert many biological functions [43]. Lipopeptides that cannot act as common antibiotics, including surfartin, iturin, and fengycin, are synthesized by *Bacillus subtilis* [44,45]. Lipopeptides are usually circular compounds with a small molecular weight of 300 to 3000 Da consisting of 7 to 10 amino acids and 13 to 17 carbon fatty acids. Surfactin has groups of β-OH on its fatty acid chain which combine with groups of -COOH to form a cyclic lactone structure. Iturins, including iturin, bacillomycin, and mycosubtilin, are types of lipopeptides that have strong antagonistic effects on fungi. There are groups of β-NH_2_ on the fatty acid chains of iturins which form amide structures. In addition, like lipopeptide, *Bacillus* can also produce other types of antibacterial substances, mainly including linear-peptide antibiotics, macrolides, phenols, polyenes, aminoglycosides, and linear polyamides [46].

## 6. Biological Functions of AMPs

AMPs have direct antimicrobial activity which depends on their ability to interact with the bacterial cell membrane or cell wall. Many AMPs exhibit direct and rapid antimicrobial activity by destroying the physical integrity of the microbial membrane via perforation or lysis or by transferring to the bacterial cytoplasm to act on intracellular targets [47,48]. Membrane interaction is believed to be a key factor in the direct antimicrobial activity of AMPs, whether AMPs combine with the membranes of pathogens or are translocated into the cytoplasm and then bind to intracellular targets [49].

### 6.1. Antiviral Activity

Some AMPs, such as α-defensins, maximins, caerin, and indolicidin, have been reported to play an important role in suppressing viral activity [50]. AMPs mainly inhibit the RNA and DNA of specific viruses with cysts, but have no inhibitory activity against those without cysts. AMPs play an antiviral role mainly by influencing the process through which viruses invade and infect the host or by directly destroying the viral envelope [51,52,53]. A direct link between the antiviral function of an AMP and its secondary structure has not yet been established, but antiviral activity varies greatly among the subclasses of an antimicrobial peptide. The specific antiviral mechanisms of AMPs include the following: (1) they interact with specific virus receptors on the cell membrane surface of host cells to prevent virus adhesion and invasion; (2) they act as a “lectin-like” agent to combine with the glycoprotein structure of the virus; (3) they change the genetic material of the virus, e.g., by interfering with RNA combination; and (4) they improve the expression level of the interferon or chemokine of the host.

### 6.2. Antibacterial Activity

#### 6.2.1. Membrane-Targeting Mechanism

**Figure 2:**
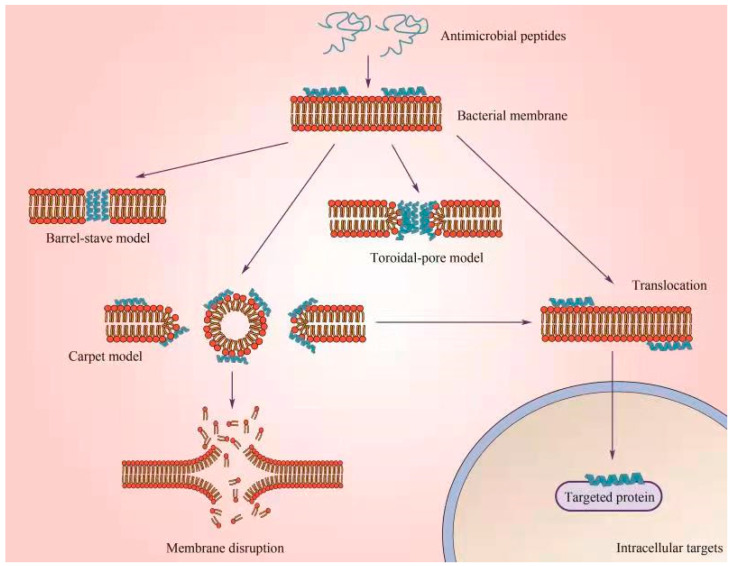
Models of the interaction between AMPs and bacterial cell membranes can be divided into the transmembrane pore model and the non-pore model. The model of the transmembrane pore hole can be further subdivided into the barrel stave hole model, toroidal hole model, and carpet model. In the barrel stave model, AMPs initially orientate parallel to the membrane but are eventually inserted vertically into the lipid bilayer to promote side-peptide and peptide interactions in a similar way to the ion channels of membrane proteins. In the toroidal pore model, the peptide is inserted vertically into the lipid bilayer to induce the local bending of the phospholipid bilayer, forming a hole composed of two peptides and the head group arrangement of the phospholipid. In addition, the AMP is absorbed parallel to the lipid bilayer and gradually reaches the threshold concentration required to cover the membrane surface, thus forming a “carpet”, which eventually leads to membrane destruction and micelle formation due to a sharp change in tension.

Most AMPs exhibit antimicrobial activity by destroying the integrity of bacterial cell membranes [54]. The inner surfaces of both Gram-positive and Gram-negative bacteria contain lipoteichoic acid (LTA) and lipopolysaccharide (LPS), which carry net negative charges to the bacterial surface, enabling an initial electrostatic attraction to cationic AMPs [55,56]. The positive charge and hydrophobic amino acid structure of AMPs allow them to selectively bind to negatively charged cell membranes of pathogens through strong electrostatic interactions [57]. After AMPs gather on the bacterial surface and reach a certain concentration, they will self-assemble and drive AMPs deeper into the bacterial membrane through interactions between the hydrophobic structural domain and the hydrophobic core of the lipid bilayer (Figure 2). Models of the interaction between AMPs and bacterial cell membranes can be divided into transmembrane pore and non-pore models [49]. The transmembrane pore model can be further subdivided into the barrel stave model and the toroidal pore model. In the barrel stave model, AMPs initially orientate parallel to the membrane but are eventually inserted vertically into the lipid bilayer to promote side-peptide and peptide interactions in a similar way to the ion channels of membrane proteins [58]. There is an assertion that the pores formed by AMPs in the phospholipid bilayer are composed of two peptides assembled with lipids in the pore model [59]. AMPs also play a role in the absence of specific holes in the membrane, reflecting a carpet model [60]. In this case, AMPs are interactive, parallel to the lipid bilayer, and gradually reach the threshold concentration required to cover the membrane surface, thus forming a “carpet”, which eventually leads to membrane destruction and micelle formation due to a sharp change in tension.

#### 6.2.2. Non-Membrane-Targeting Mechanism

Non-membrane-targeted AMPs can be divided into two categories, including AMPs targeting bacterial cell walls and intracellular targets. Similar to conventional antibiotics such as penicillin, AMPs also inhibit the cell wall synthesis of pathogens. AMPs usually interact with various precursor molecules required for cell wall synthesis. One of the primary target molecules is the highly conserved lipid II [61]. Human defensins can selectively bind to lipid II to block the biosynthesis of the bacterial cell wall, resulting in pathogen depth [62,63]. In addition, many AMPs can be translocated into the cytoplasm through the cell membrane and achieve their antimicrobial activity by targeting key cellular physiological processes [64]. The main mechanisms through which AMPs flow into the cytoplasm to inhibit the activity of pathogens include the suppression of the synthesis of proteins and nucleic acids, the destruction of enzyme activity, the inhibition of cell respiration, the induction of the production of active oxygen free radicals, and the activate leakage of adenosine triphosphate (ATP) and nicotinamide adenine dinucleotide (NADH) via mitochondrial destruction [58,65]. For example, the proline-rich AMPs known as apidaecins and oncocins from insects inhibited protein translation via 70S ribosomes [36].

### 6.3. Antifungal Activity

The antifungal action of AMPs may function to inhibit or kill fungi by interfering with the synthesis of fungal cell walls or enhancing fungal cytolysis. Some antifungal peptides extracted from plants are rich in polar and neutral amino acids, which may relate to the antifungal functions of antibacterial peptides [20,66]. The antifungal mechanisms of AMPs function to (1) interfere with or inhibit the synthesis of fungal cell walls; (2) interact with the fungal cell membrane and damage the structure and function of the membrane; (3) cause fungus mitochondria to vacuolize, which inhibits the respiration of the fungus; and (4) act on fungal enzymes, nucleic acids, and other biological macromolecules to influence metabolic processes.

### 6.4. Antiparasitic Function

Parasitic diseases are a great threat to livestock, poultry, and human health. Because of the increasing drug resistance of parasites and the toxicity of antiparasitic drugs, parasitic diseases cause negative influences on human nutrition. Some AMPs, such as α-defensin, scorpine, and phylloseptins, can be used to treat parasitic infections. Some AMPs, such as BMAP-18, can prevent the spread of parasites [67]. Negatively charged parasite membranes cause AMPs to accumulate and be inserted into them. AMPs that cross the cell membrane of the parasite cause damage that leads to a pH imbalance between the inside and outside of parasite cells, causing an outflow of intracellular ions and macromolecules [68]. A marine synthetic AMPs inhibits Trichomonas vaginalis by destroying the membrane [69]. The peptide Jellein derived from bee royal jelly has shown a significant effect on the Leishmania parasite [70]. Some AMPs can also enter a parasite’s cells to increase the intracellular Ca^2+^ concentration or destroy the mitochondrial membrane potential to prevent the production of ATP, which kills the parasites [68]. However, it is worth noting that some AMPs are toxic to human cells due to their disruption of cell membranes, which would limit their application in curing parasitic diseases.

### 6.5. Antineoplastic Activity

**Figure 3:**
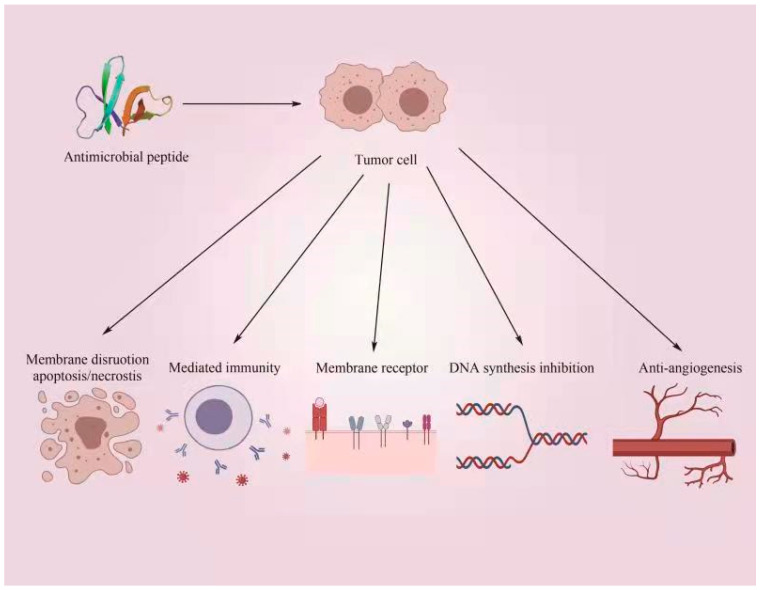
Mechanism of the anticancer activity of AMPs. Antitumor mechanisms of AMPs include the destruction of the tumor cytoskeleton, the destruction of the tumor cell membrane, the injury of the mitochondrial membrane of tumor cells, the induction of the apoptosis or necrosis of tumor cells, the enhancement of immunity, the inhibition of peripheral angiogenesis of tumors, and the inhibition of DNA synthesis to affect the tumor cell cycle and inhibit growth, respectively.

Some AMPs have excellent antitumor functions, providing a new method for exploring drugs for treating tumors. Most AMPs reported to have antitumor effects are extended AMPs [20]. According to the targets of antibacterial peptides, antitumor antibacterial peptides can be divided into two categories, whereby one of which is composed of antibacterial peptides that only fight against microorganisms and tumor cells while having no effect on healthy mammalian cells, such as silkworm peptides and frog skin peptides (Figure 3). The other class of AMPs, which includes *Lasioglossins* extracted from bee venom and the defense proteins (HDPs) of human neutrophils, is toxic to microorganisms, tumor cells, and even normal mammalian cells. The antitumor mechanisms of AMPs include the destruction of the tumor cytoskeleton, the destruction of the tumor cell membrane, the injury of the mitochondrial membranes of tumor cells, the induction of the apoptosis or necrosis of tumor cells, the enhancement of immunity, the inhibition of peripheral angiogenesis of the tumor, and the inhibition of DNA synthesis to affect the tumor cell cycle and inhibit growth [71]. However, we cannot predict whether AMPs will have antitumor effects from their structure.

### 6.6. Regulation of the Host Immune System

#### 6.6.1. Regulation of Pro-Inflammatory and Anti-Inflammatory Immune Responses

**Figure 4:**
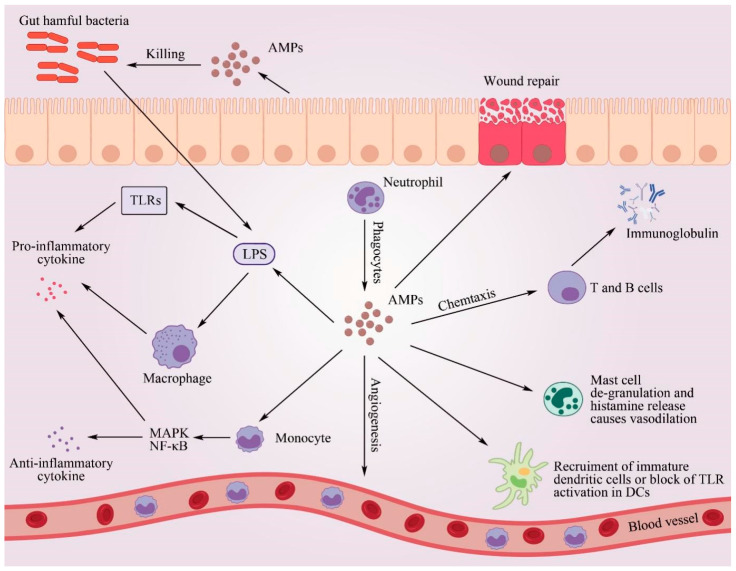
Mechanisms of AMPs improving host immune function. AMPs can inhibit inflammatory responses caused by Toll-like receptor ligands. Antimicrobial peptide inhibits an increase in the pro-inflammatory cytokine TNF-α from macrophages induced by LPS via Toll-like receptor ligands. This prevents a combination of LPS and lipopolysaccharide-binding proteins to suppress the increase in inflammatory cytokine levels caused by LPS on the cell membranes of macrophages. Furthermore, AMPs mediate NF-κB signaling and cellular immune responses by regulating the MAPK signaling pathway to improve immune function.

AMPs can inhibit inflammatory responses caused by Toll-like receptor ligands [72]. Inflammation and sepsis are always caused by LPS located on the cell walls of Gram-negative bacteria (Figure 4). Recombinant antimicrobial peptide microcin J25 also provides barrier protection by preserving structural integrity and reducing inflammatory infiltrates of the colon epithelium [73]. The antimicrobial peptide clavanin inhibits an increase in the pro-inflammatory cytokine TNF-α from macrophages induced by LPS [74]. AMPs, such as the wild silkworm CP29, melittin hybrid cationic AMPs, and slime mold element B, can prevent a combination of LPS and lipopolysaccharide-binding protein, resulting in the suppression of an increase in inflammatory cytokine levels caused by LPS on the cell membranes of macrophages [7,75]. The antimicrobial peptide LL-37 regulates TLR-mediated inflammatory responses and inhibits elevated levels of proinflammatory mediators caused by LPS and lipoteichoic acid [76]. The antimicrobial peptide LL-37 regulates TNFAIP2 genes, activates nuclear-factor-k-gene binding (NF-κB1) P105/P50, and also mediates cellular immune responses by regulating the mitogen-activated protein kinase (MAPK) signaling pathway along with glyceraldehyde phosphate dehydrogenase in human monocytes [77]. Overall, AMPs regulate different cellular responses through MAPK and NF-κB, controlling the production of pro-inflammatory cytokines (IL-1β) and anti-inflammatory cytokines (IL-10) and thus achieving a balance between inflammatory and inhibitory responses. Although antibiotics can kill pathogens, they cannot neutralize LPS on the cell membrane. Therefore, when pathogenic microorganisms release substrates derived from the cell walls of pathogens after antibiotic treatment, the inflammatory responses are exacerbated. In addition, studies have shown that AMPs can directly act on B cells and T cells to regulate the acquired immune functions of the host. In mice, AMPs inhibit the production of IL-4 by the host T cells and regulate the production of immunoglobulin G1 (lg G1) by B cells [78]. Studies have shown that synthetic defensin can promote the proliferation of T lymphocytes in the spleen, lamina propria, and lymph nodes, resulting in an improvement in the host’s immunological function [79].

#### 6.6.2. Chemotaxis Function

Another important immunological function of AMPs is the regulation of chemotaxis, which stimulates the migration of white blood cells to the site of infection [80]. AMPs can enhance chemotaxis in both direct and indirect ways. AMPs indirectly enhance the chemotaxis of immune cells, including mononuclear macrophages, epithelial cells, mast cells, and T lymphocytes, by promoting the expression of the chemokines CXCL8/IL-8 and CCL2/McP-1 [81,82]. When the concentration of AMPs is high, host defense peptides can directly serve as chemokines to recruit various immune cells and epithelial cells to the site of infection in order to enhance bacterial clearance and promote repair following cell damage. The mechanism through which the antimicrobial peptide IDR-1002 modulates the chemotaxis of white blood cells appears to be mediated by G-protein-coupled receptors (GPRs), phosphatidylinositol 3-kinase (PI3K), NF-κB, and MAPK signaling pathways [83].

#### 6.6.3. Enhanced Ability of the Host Immune System to Kill Harmful Bacteria

AMPs can kill pathogenic microorganisms directly or indirectly via other innate immune mechanisms, such as neutrophil-derived extracellular trap nets (NETs) and master-derived extracellular trap nets (MCETs). NETs and MCETs are extracellular network structures composed of cellular DNA and AMPs, such as LL-37 and host defense peptides [84]. The formation of extracellular network structures is triggered by pathogenic microorganisms, activated platelets, and different immune stimulation signals. Invading bacteria are trapped in extracellular traps by proteins, such as myeloperoxidase, which are released via neutrophil degranulation [85]. The roles of AMPs in NETs and MCETs remain unclear, but AMPs have recently been shown to inhibit the formation of cell membranes, a function which is similar to the selective destruction of cell membranes of pathogens by AMPs in NETs [86]. AMPs can enhance the intracellular bactericidal action of neutrophils and macrophages. Synthesized cationic AMPs of innate defense regulators (IDRs) enhanced the ability of neutrophils to kill *Escherichia coli* (*E. coli*) and promoted neutrophils to release the host defense peptide LL-37 and defense hormone [83]. The antimicrobial peptide HLF1-11 significantly enhanced the bactericidal ability of macrophages against Staphylococcus aureus [78]. This may be due to the formation of superoxide free radicals or the activation of macrophages and neutrophils by cytokines [87].

## 7. Factors Affecting the Biological Functions of AMPs

The biological functions of AMPs that work to suppress pathogens and improve host immunology are related to peptides’ molecular structures and electric charges, as well as the hydrophobicity of their amino acids.

### 7.1. Conformation of AMPs

The composition of amino acids directly affects the structural formation of AMPs by changing their helicity. The substitution of L-type amino acids with D-type amino acids results in difficulty in forming a helical structure among AMPs, thus reducing the helical contents of the system [88]. Many reports have demonstrated that using D-type amino acids to replace L-type amino acids in AMPs reduced their antibacterial activity [89,90]. This result shows that the high degree of helicity is an important reason for the antimicrobial and hemolytic activities of AMPs.

### 7.2. Electrical Charge Number of AMPs

Bacterial membranes are rich in phospholipid molecules; therefore, bacteria have a negative charge on their surfaces. The electrostatic interaction between AMPs and the bacterial membrane directly determines whether or not AMPs can form pores on the bacterial cell membrane and further destroy the bacterial cell membrane. Therefore, if AMPs contain more positively charged amino acids, the connection between AMPs and bacteria becomes stronger [91]. The lipopolysaccharides and phosphoric acid of bacteria increase the number of negative charges on the bacterial surface. Antibacterial peptides can recognize specific sites or molecules on the cell membrane of pathogens due to the specific selectivity of antibacterial peptides [92]. In addition, due to a difference in isoelectric points, amino acids can have different charges under different pH conditions [93]. It is worth noting that the antibacterial activity of AMPs is influenced by the number and structure of positively charged amino acids in the peptide chain. However, if AMPs carry excessive positive charges, the electrostatic repulsion among AMPs may be too great, exceeding the electrostatic attraction between the AMP and bacterial cell membrane. In this situation, the enrichment of AMPs on the bacterial membrane surface cannot be achieved; thus, pores are not formed, and the antibacterial activity is reduced [94].

### 7.3. Hydrophilic and Hydrophobic Properties of AMPs

Amphiphilicity is an important structural characteristic of an AMP. AMPs demonstrate hydrophobicity when their content of hydrophobic amino acids is high. The hydrophobicity of antibacterial peptides alters the helix structure of the peptide chain, which changes the antibacterial activity [95]. The antibacterial activity of AMPs is the result of interactions between hydrophilic groups and hydrophobic groups after the formation of the secondary structure. Hydrophobicity plays a key role in allowing AMPs to pass through bacterial membranes, since the antimicrobial activity of antibacterial peptides can be regulated by changing the content and position of each hydrophobic amino acid in the antibacterial peptide [22]. However, with increased hydrophobicity, there is a potential risk. Studies have shown that an increase in the hydrophobic amino acid content of antibacterial peptide chains improves peptides’ hydrophobicity and antibacterial activity but also increases the toxicity of AMPs to mammalian cells [56]. Furthermore, the presence of hydrophobic groups reduces the solubility of antibacterial peptides, leading to polymer formation in solution, which reduces antibacterial activity and promotes hemolytic activity.

## 8. Biological Expression Systems for AMPs

Natural AMPs derived from animals and plants are difficult to extract and purify. Synthetic AMPs have a lower yield but are more expensive compared with those produced by animals and microflora. In recent years, the recombinant expression of AMPs via genetic engineering technology has been shown to be of great significance for the commercial production of AMPs. The common expression systems for AMPs are mainly divided into prokaryotic expression and eukaryotic expression systems. The advantages of prokaryotic expression are its short expression periods, high expression quantity, and low expression cost, but problems with lack of modification and difficulty may arise when purifying AMPs. The advantages of eukaryotic expression are the facts that there is no toxicity to host cells and extracellular expression makes purification easy. The disadvantages of eukaryotic expression are its high expression cost and long expression periods.

### 8.1. Escherichia coli Expression System

*Escherichia coli* expression is a widely used prokaryotic expression system in the field of genetic engineering to industrialize antimicrobial peptide production [96]. Today, it has a mature commercial expression vector and gene operating system. However, AMPs cannot be directly expressed using the *E. coli* system in commercial settings. AMPs are highly lethal to host *E. coli*, and the expression of AMPs in *E. coli* inhibits the proliferation of the host, which reduces the expression of AMPs. In addition, antibacterial peptides are very sensitive to proteases and can be easily degraded by proteases secreted by the host *E. coli*; therefore, it is difficult to achieve high expression. The fusion expression of AMPs in *E. coli* could effectively solve the above problems [97]. Recombinant fusion proteins can be further purified to obtain expression targets with antibacterial activity. The commonly used carrier proteins are those that promote soluble expression and precipitation expression [9].

Carrier proteins that promote soluble expression, such as glutathione transferase (GST) and thioxoreductase (TRX), promote the expression of the target protein in the host in a solubilized and correctly folded manner [98,99]. The small-molecule ubiquitinated protein (SMUP) is a new expression vector which can increase the proportion of the target protein in the fusion protein and improve the yield of the target protein. The specificity of SMUP protease makes it easier for AMPs to be released from the fusion protein [100]. More importantly, soluble expressed fusion peptides are easier to purify and can be directly purified with a specific affinity using six histidine tags or GST tags.

Carrier proteins that improve precipitate expression promote fusion proteins to form inclusion bodies in the host, such as ketosteroid isomerases [101]. Inclusion bodies are water-insoluble structures formed by large amounts of proteins in *E. coli* gathered and formed in a membrane-enclosed or membrane-leakless structure. Inclusion bodies can be collected in large quantities via simple fractional centrifugation, and their purification process is simple. However, expressed AMPs may not fold correctly, resulting in a lack of biological activity [9]. Therefore, the activity of expressed AMPs must be activated via renaturation, which leads to a limited application scope of precipitation expression carriers.

### 8.2. Yeast Expression Systems

In practice, *Saccharomyces cerevisiae* and *Pichia pastoris* are commonly used to express AMPs. Studies on the genotypes and vectors of *Saccharomyces cerevisiae* are relatively mature; however, it is not often used as an expression host because of its high consumption of carbon sources during fermentation [102]. *Pichia pastoris* is suitable as an expression host and has commercial expression carriers. Human defensin LL-37 and β-defensin 2 have been successfully expressed in large quantities in *Pichia pastoris* [103]. Compared with the *E. coli* expression system, the main advantages of the recombinant expression of AMPs in yeast are as follows: (1) some AMPs have no toxicity to eukaryotic cells, and yeast can directly express the targeted AMPs [104]; (2) yeast can secrete AMPs into the extracellular space, which can avoid a step of bacterial fragmentation and is beneficial for the purification of targeted proteins [105]; (3) eukaryotic expression has a modification function, being suitable for some complex AMPs, such as those rich in disulfide bonds and that undergo glycosylation [103]. Unlike *E. coli*, yeast cell walls have no endotoxin and are rich in nutrients; therefore, AMPs expressed by yeast can be added to the rations of livestock as new additives. However, compared with the *E. coli* expression system, the disadvantages of yeast expression systems are their comparatively lower expression efficiency, longer fermentation culture cycle, and higher expression cost.

### 8.3. Plant Expression Systems

In recent years, many researchers have introduced the genes of AMPs into plants, such as corn and soybean, via transgenic technology, in order to cultivate pathogen-resistant plants by improving the resistance of transgenic plants to microorganisms [106,107]. Due to the antibacterial properties of AMPs, the use of microorganisms as hosts limits the functional expression of AMPs to some extent; thus, plant expression systems may be a good substitute. However, only a few studies have targeted the extraction of AMPs from plants as expression hosts. As superior eukaryotes, plants are suited to the expression of AMPs with complex disulfide bond structures, such as cyclotides containing six cysteines. However, the yield of AMPs from plant expression is difficult to control. In addition, there are specific glycosylation pathways in plants which are different from those in animals. The expression of animal AMPs may alter their original structure and lead to a loss of antimicrobial activity. At present, the use of plants as expression carriers of AMPs is still in development. Recently, *Chlamydomonas reinhardtii* was found to be a relatively suitable host for the expression of AMPs because it has a homologous recombination pathway, which can reduce glycosylation in superior plants [108]. However, a mature expression system using plants to produce AMPs requires further study.

## 9. Current Questions regarding the Application of AMPs

AMPs have antibacterial and immune modulatory activities, and bacterial pathogens cannot readily develop resistance to them. These advantages confer broad application prospects regarding peptides, but there are still some problems related to the practical application of AMPs.

### 9.1. Sources of AMPs

At present, the preparation methods for antibacterial peptides mainly include biological extraction, chemical synthesis, and genetic engineering expression. Although AMPs can be produced widely in nature, the extraction and purification processes are difficult, and they have the limitations of a low yield, difficult isolation technology, and high extraction cost. The preparation of AMPs via biological extraction methods cannot produce a sufficient quantity to meet the needs for commercial application, and the artificial synthesis of AMPs using chemical methods is very expensive [78]. In addition, with the biological expression of AMPs via genetic engineering technology, it is difficult to obtain targeted products and high expression efficiency.

### 9.2. Toxicity of AMPs to Host Cells

Another factor inhibiting the use of AMPs is their potential toxicity to mammalian cells. The antimicrobial peptide LL-37 can induce mast cells to release histamine, which, in turn, increases cell permeability and causes some pathological reactions [109]. Mice injected with a high concentration of the antimicrobial peptide LL-37 were developed erythema and the characteristics of inflammatory rosacea [110]. The mechanism of the cytotoxicity of AMPs remains unclear, and further studies should be conducted to elaborate upon these potential actions in the future.

### 9.3. Stability of AMPs

Another factor affecting the application of AMPs is the fact that the stability of AMPs in vivo is generally low because they are easily degraded by the endogenous digestive enzymes of the host. In the human digestive tract, trypsin and chymotrypsin can break the basic amino acid (arginine and lysine) and hydrophobic amino acid (tryptophan and phenylalanine) residues of AMPs, eliminating their antimicrobial activity. AMPs are also easily degraded by enzymes secreted by pathogenic microorganisms. For example, metalloproteinases secreted by Staphylococcus aureus can inactivate AMPs such as LL-37. Therefore, researchers have proposed many protocols to prevent the degradation of AMPs in vivo, including the synthesis of AMPs using D-type non-natural amino acids, modifying the ends of antimicrobial peptide chains via acetylation and amidation, and the cyclization of AMPs [111,112]. However, the strategies mentioned above significantly increase the production cost of AMPs. In addition, it is not clear how D-amino acids act on target proteins and whether D-amino acids affect receptor–ligand interactions to regulate immune responses and functions [78].

## 10. Conclusions

Antimicrobial peptides are potential substitutes for antibiotics due to their positive biological functions in the host and the difficulty in generating resistance genes. However, there are still some limitations in the production and application of antimicrobial peptides in various disciplines and commercial applications. Novel technologies, such as mature genetic engineering and purification methods, should be further developed to increase the yield of antimicrobial peptides. Furthermore, the actions of antimicrobial peptides in destroying cell walls and membranes to kill pathogens have become widely recognized; however, the relationships between antimicrobial peptide structures and their antimicrobial activity and mechanisms in regulating the immune function of the host need to be further elucidated.

## Figures and Tables

**Table 1:** Comparison of prokaryotic AMPs, eukaryotic AMPs, and antibiotics.

Property	Eukaryotic AMPs	Prokaryotic AMPs	Antibiotics
Biological synthesis	Ribosome	Ribosome, modification	Non-ribosome
Spectrum	Selective	Narrow	Broad
Host cell immunity	No immunity	No immunity	No immunity
Targets	Multiple	Multiple	One or a class
Resistance	Not easy	Difficult	Easy
Stability	Unstable	Moderately stable	Stable
Bioactivity	High	Low	Very high
Production cost	Very high	High	Low

## Abbreviations

ATP: Adenosine triphosphate; Cys, Cysteine; GPRs, G-protein-coupled receptors; GST, Glutathione transferase; Ig G, Immunoglobulin G; LPS, Lipopolysaccharide; LTA, Lipoteichoic acid; MAPK, mitogen-activated protein kinase; MCETs, master-derived extracellular trap nets; NETs, neutrophil-derived extracellular trap nets; NF-κB, nuclear-factor-k-gene binding; PI3K, Phosphatidylinositol 3-kinase; SMUP, small-molecule ubiquitinated protein; TLRs, Toll-like receptors; TRX, Thioxoreductase.
